# Analyzing the effect of interview time and day on emergency medicine residency interview scores

**DOI:** 10.1186/s12909-022-03388-6

**Published:** 2022-04-26

**Authors:** Alanna O’Connell, Sean Greco, Tingting Zhan, Tracy Brader, Megan Crossman, Robin Naples, Anthony Sielicki, Megan Stobart Gallagher, Peter Tomaselli, Dimitrios Papanagnou

**Affiliations:** 1 Thomas Jefferson University Hospital, 111 S 11th St, Philadelphia, PA 19107 United States; 2 Carolinas Healthcare System, 1000 Blythe Blvd, Charlotte, NC 28203 United States; 3 Einstein Medical Center Philadelphia, 5501 Old York Rd, Philadelphia, PA 19141 United States

**Keywords:** Residency interviews, Residency match, Medical student interviews

## Abstract

**Background:**

When it comes to scheduling interviews, medical students may wonder if they need a strategy to increase their likelihood of matching. Previous studies examined the temporal effects of the residency interview on overall match rate; however, there are additional factors that affect the match process, including board examination scores and letters of recommendation. Only few studies have examined the effect interview time of day has on match success. The current study examines the impact date and time of interview during the interview season have on candidates’ respective interview scores.

**Methods:**

Interview data over a three-year period (i.e., three interview cycles) was examined at a PGY-1-3, ACGME-accredited EM residency program in Philadelphia. Date of interview and time of day of interview (i.e., morning versus afternoon) was examined. A linear regression analysis was performed to determine if there is a statistically-significant difference in overall interview scores based on date during the interview season and time of day.

**Results:**

There is no statistically-significant effect of time of day or date on residency interview scores.

**Conclusions:**

Our findings are congruent with other studies on the temporal effects of residency interviews on overall match rate. Findings should provide reassurance to students scheduling interviews, as time slots have not been found to have a significant relationship with overall interview score. Future studies should more holistically analyze the residency application process.

## Introduction

When it comes time to schedule interviews, medical students may wonder if they require a strategy for optimally timing their interviews to increase their likelihood of successfully matching in Emergency Medicine. There has been some previous research into the temporal effect of interviewing on match success, but these studies mostly evaluated general time frame and match success rate, and did not necessarily include time of day or look at standalone interview scores. This study further evaluates this question, and examines whether time of day or date during the interview season has any influence on overall interview score, not just match rate.

The residency match process is perhaps one of the most stressful milestones of medical school, particularly the residency interview. Following an initial screening process, students are invited to interview with residency programs in their medical specialty of choice. This process has become increasingly stressful for applicants pursuing emergency medicine (EM) as the number of applications has increased substantially [1]. Interviews are typically scheduled from October through January of the fourth-year of medical school, leading many applicants to question the timing of their interviews and the impact interview timing will have on their respective rank-list position for specific programs as they enter the match [2]. During interviews, students are typically scored according to a rubric for key factors and skills programs value [3, 4]. These scores have the potential to impact a candidate’s position on a program’s rank list, which is then submitted to the National Residency Matching Program (NRMP).

Several studies have examined the timing of residency interviews and the likelihood to match; no statistically-significant relationships were noted in these studies. Studies have been performed in internal medicine [5], anesthesia [6], emergency medicine [7] and a larger review of multiple specialties [8]. These studies tended to group interview dates into broad categories of early, middle, and late interviews, and then determined if there were any correlations to the residency match. These studies found no effect on interview timing and residency match rate. However, these studies did not look at time of day as an independent variable (i.e., morning versus afternoon), nor did they examine interview scores assigned to applicants. Previous studies have only commented on global scores and overall match rate.

Additionally using match rate as a proxy for interview performance introduces several confounding factors. There are many other data points involved in the match such as board examination scores, letters of recommendation, and personal statements. Each of these can independently influence a candidate’s match outcome [9, 10]. Previous studies [5–8] of the temporal effects on residency interviews did not examine interview scores assigned to applicants; these effects may, in fact, represent a better understanding of the temporal effects of interview timing given that it removes several of the aforementioned confounding factors.

Temporal effects have been extensively studied in non-medical fields, such as law, and have contributed valuable findings to the impact of temporal effects on discrete outcomes of interest. Danzinger et al. looked at Israeli parole board rulings, which defined favorable rulings (i.e., those in which parole was granted) and unfavorable rulings (i.e., those in which a judge denied parole or decided to reconvene at a later date). They found that within one session of hearing cases, the ratio of favorable rulings decreased from about 65% to close to zero. After a food break, however, this ratio reset to close to the original 65% [11]. Many residency programs have two interview sessions on their interview days - one in the morning and one following lunch [12]. The principle demonstrated by Danzinger could potentially introduce a flaw in the interview format, assigning more favorable scores to interviewees based on when their interview took place.

The authors of this retrospective study sought to examine if there was any relationship between either time of day, or date in the interview season, and candidates’ interview scores. We hypothesized that overall interview score would not be affected by either time of day or date of interview given previous observations.

## Methods

The study was approved by the institutional review board (IRB) at our institution. We examined data from an academic, PGY-1 through − 3 Emergency Medicine (EM) residency program accredited by the Accreditation Council of Graduate Medical Education (ACGME). The program is located at a Level-1 trauma center in a large urban city. Interview data for the three interview cycles (i.e., 2016-2017, 2018-2019, and 2019-2020) was reviewed. Candidates invited to interview at our program typically interview with 4 to 5 faculty members, two of which include the program director and the associate program director, for a brief interview. Variation in faculty was typically year to year although occasionally a member of the core group had to be replaced by a different EM faculty member due to scheduling conflicts. Faculty members were typically experienced at residency interviewing. All of the faculty were Emergency Medicine residency trained and held faculty appointments through through the associated medical school. Interviews are structured and approximately 15 min in duration in either the morning or afternoon. The morning interview session typically ran from 9 am -noon and the afternoon session typically ran from noon to 3 pm on interview days. The interview sessions are assigned and students do not get to select a preference for interview session. While there are no breaks built into this schedule, the afternoon session applicants are getting tours/food with residents while the morning session applicants are interviewing and vice versa. During this study period two of the interviewers were consistent with changes in the other 2 to 3 interviewers depending on the year. Following the interview, a debriefing is held among interviewing faculty to discuss candidates’ interview performance, after which a score sheet is filled out by the program director with the date, time, and interview score. Interview candidates are given a global interview score that is based on candidates’ perceived compassion, interpersonal skills, maturity, and overall gestalt in comparison to peers. Individual interviewer scores were not available for analysis however the global score was created from an additive composite score with each interviewers score weighted equally. An objective scoring format derived from the Likert Scale was used in the 2018-2019 and 2019-2020 interview cycles, allowing for more consistent scoring. In addition, the minimum number of students interviewed in a cycle was 127. The minimum global score was 0 with a maximum score of 25.

**Fig. 1:**
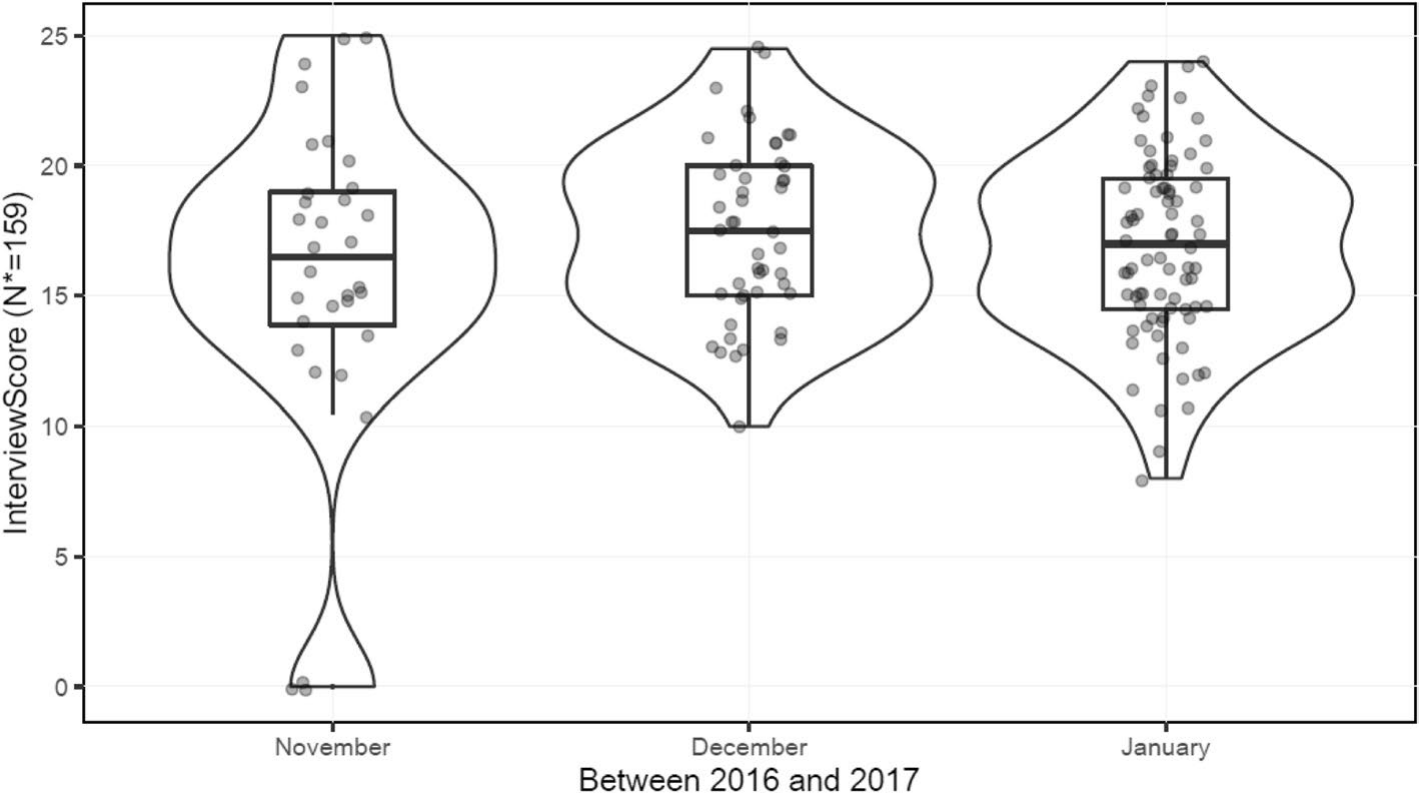
2016-2017 Interview Cycle

Student-identifying data was removed from the interview score sheets. Only interview raw scores were entered into a data bank with the interview date and time. Time was placed into two categorical variables, which represented morning or afternoon interviews. Data points were grouped according to date and time, with subsequent interview score recorded adjacently. A statistical analysis was performed by fitting a multivariable ordinary least squares model using R Core Team software [13]. Backward stepwise variable selection for the multivariable model was based on Akaike Information Criterion (AIC). Further analysis was then performed using R package MASS software [14]. Linear contrasts were then constructed using R package multcomp [15] to ultimately review the data and create Fig. 1 through 3, which are discussed in the Results. All methods were performed in accordance with the relevant guidelines and regulations.

## Results

We reviewed a total of 453 interviews from 2016 to 2020 at our institution. Of these, two of the interview cycles followed the same format with the exception of the 2016-2017 interview cycle. There were 159 Emergency Medicine residency interviews in the 2016-2017 interview cycle. This cycle used a different scoring format- the rubric was the same but the score sheets used a different format and so the time of day the interview occurred was not recorded. A Likert scale was also not utilized this year. Additionally there were no interviews in October that year. Therefore, this data was analyzed separately, and we only considered the effect date of interview may have had on the interview score. These findings are depicted in Fig. 1. There was no statistically significant relationship between date and the interview score for the 2016-2017 cycle.

**Fig. 2:**
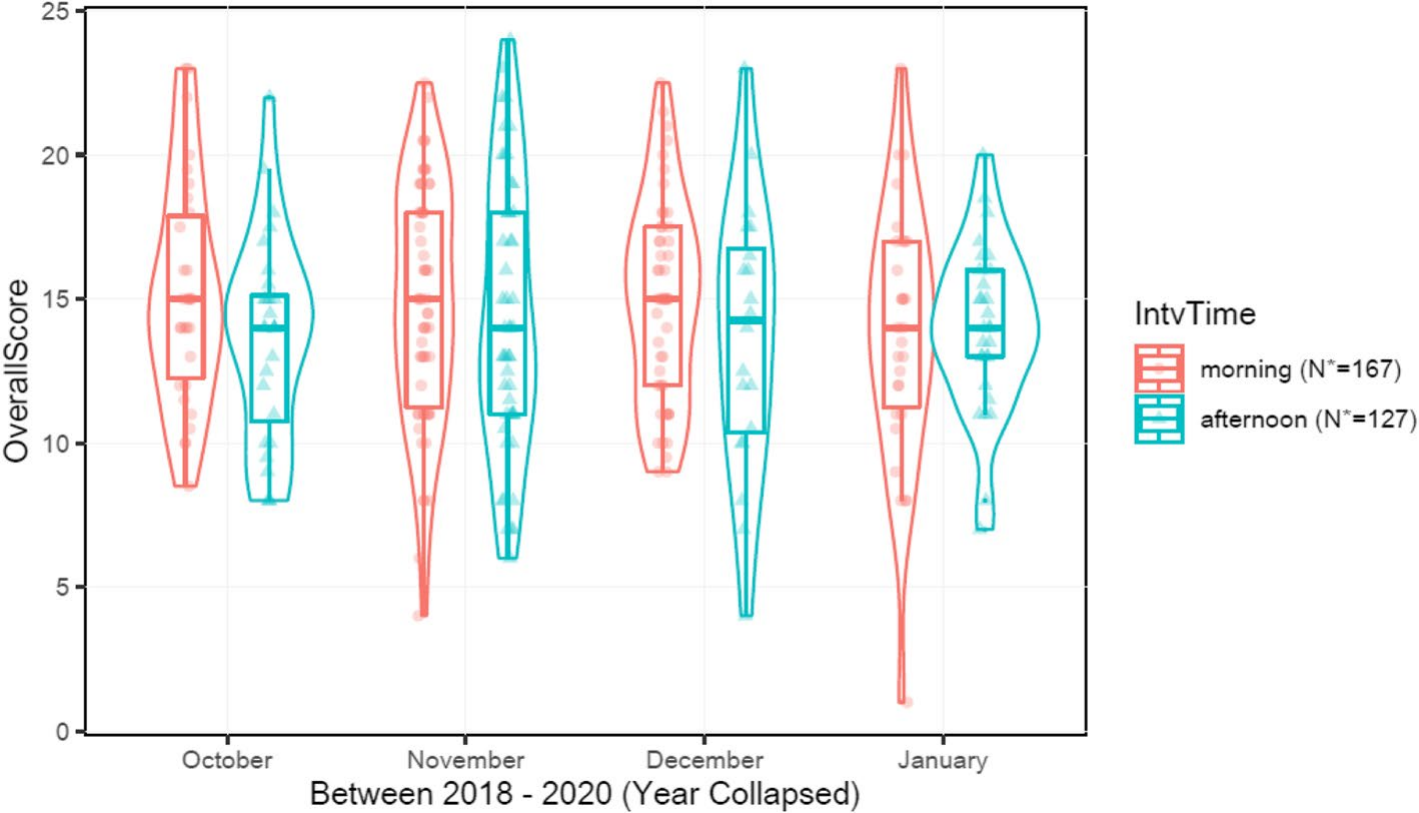
2018-2019 and 2019-2020 Interview Cycles (Summary)

There were a total of 294 EM residency interviews in the 2018-2019 and 2019-2020 interview cycles. These cycles both used the same scoring format and recorded interview time; therefore, they were analyzed in aggregate. Figure 2 summarizes the data from each cycle by month. During the month of December 2019, afternoon interviews were not offered. There were no statistically-significant temporal relationships observed between the date, time, and the interview score for 2018-2019 and 2019-2020 cycles.

## Discussion

Our findings correlate with other similar studies performed across graduate medical education. By using a more objective interview outcome measure with similar findings, our study provides more validity to the findings of these prior studies. These observations confirm our initial hypothesis that date and time of interview are not correlated with interview score. This should reassure students that the timing of their interview will have no effect on its outcome. It should also demonstrate to residency programs that temporal bias does not play a significant role in the interview process. An interesting consideration on review of the data is that while most of the scores clustered in the middle of the range, if the outliers also change temporally. Future studies may want to look into if these more extreme scores are from a halo or horn effect. Some reasons why our process specifically does not seem to have a temporal bias may include the rubrics used for creating our interview scores which tend to standardize the interview impressions among faculty. Additionally, keeping the majority of our interview faculty consistent throughout the process likely helped to keep scores standardized as well. Another factor may have been the structure of the interview day itself, where faculty members take the whole day to do interviews without a prolonged lunch break (which was shown to create some bias in the Danzinger study (11)). Having all interviewers be EM trained faculty members may have helped to alleviate temporal bias. Using similar measures of consistent EM trained faculty members with consistent rubrics without a prolonged break between sessions may help to reduce this at other institutions.

### Limitations

A major limitation of this study relates to data availability. This represents findings from a single residency program. Furthermore, the system of scoring interviews at our program may vary from scoring processes at other institutions. Interview scores from only three interview cycles were able to be obtained; and the scoring worksheet from the 2016-2017 cycle was slightly different from other years in that the timing of morning/afternoon was excluded the Likert scale was not present.

The minimum number of students interviewed in a cycle was 127. This created a larger pool of scores in each cycle, which allowed for a more reliable analysis. If this study were to be reproduced, interview scores from more than 3 cycles should ideally be included. Having access to more consistent information from additional interview cycles would increase the scope and generalizability of our findings.

Also as reported above while there was an effort to keep the faculty interviewers consistent throughout the study there was some variation of faculty members through the years which may have impacted the data. Additionally it was not tracked which students interviewed with the 4 faculty cohort versus the 5 faculty cohort which may have influenced the scores.

There was also some variability in scores from the 2018-2019 year to the 2019-2020 years- the reason for this is unclear. It is possible that with time, interviewers became more comfortable/standardized in their approach to scoring or due to faculty variation (specific data on interviewers for each session is unavailable).

An additional limitation is the current change to a virtual interview format following the COVID-19 pandemic. This study was performed pre-pandemic and the new virtual format has many differences from the traditional in person interview. To see if our findings are consistent in the new format a new study done with data from virtual interviews should be performed.

## Conclusions

While other studies looking at the temporal effects of residency interview date and time on match outcome have been described, we did not find any statistical difference in these relationships. Our study addressed the notion that there are confounding factors that inform students’ match outcomes, which cannot be directly attributable to the timing of their interview. Our study did not observe any statistical difference in timing of an individual’s interview and their score. Findings should reassure both students and interviewers that a particular interview slot should not put them at a disadvantage in the match process.

## Data Availability

The datasets generated during and analyzed during the current study are not publicly available due to containing medical student demographics but a de-identified version is available from the corresponding author on reasonable request.
